# Diagnosis and Management of Atrial Fibrillation in Acute Ischemic Stroke in the Setting of Reperfusion Therapy: Insights and Strategies for Optimized Care

**DOI:** 10.3390/jcdd10110458

**Published:** 2023-11-12

**Authors:** Jay Patel, Sonu M. M. Bhaskar

**Affiliations:** 1 Global Health Neurology Lab, Sydney 2150, Australia; 2 South Western Sydney Clinical Campuses, UNSW Medicine and Health, University of New South Wales (UNSW), Sydney 2170, Australia; 3 Ingham Institute for Applied Medical Research, Neurovascular Imaging Laboratory, Clinical Sciences Stream, Sydney 2170, Australia; 4 NSW Brain Clot Bank, NSW Health Pathology, Sydney 2170, Australia; 5 Department of Neurology & Neurophysiology, Liverpool Hospital, South Western Sydney Local Health District (SWSLHD), Sydney 2170, Australia; 6 Department of Neurology, National Cerebral and Cardiovascular Center (NCVC), Suita 564-8565, Osaka, Japan

**Keywords:** stroke, atrial fibrillation, reperfusion therapy, thrombectomy, thrombolysis

## Abstract

Reperfusion therapy in the form of intravenous thrombolysis (IVT) and endovascular thrombectomy (EVT) has revolutionised the field of stroke medicine. Atrial fibrillation (AF) patients constitute a major portion of the overall stroke population; however, the prevalence of AF amongst acute ischemic stroke (AIS) patients receiving reperfusion therapy remains unclear. Limitations in our understanding of prevalence in this group of patients are exacerbated by difficulties in appropriately diagnosing AF. Additionally, the benefits of reperfusion therapy are not consistent across all subgroups of AIS patients. More specifically, AIS patients with AF often tend to have poor prognoses despite treatment relative to those without AF. This article aims to present an overview of the diagnostic and therapeutic management of AF and how it mediates outcomes following stroke, most specifically in AIS patients treated with reperfusion therapy. We provide unique insights into AF prevalence and outcomes that could allow healthcare professionals to optimise the treatment and prognosis for AIS patients with AF. Specific indications on acute neurovascular management and secondary stroke prevention in AIS patients with AF are also discussed.

## 1. Introduction

**Figure 1:**
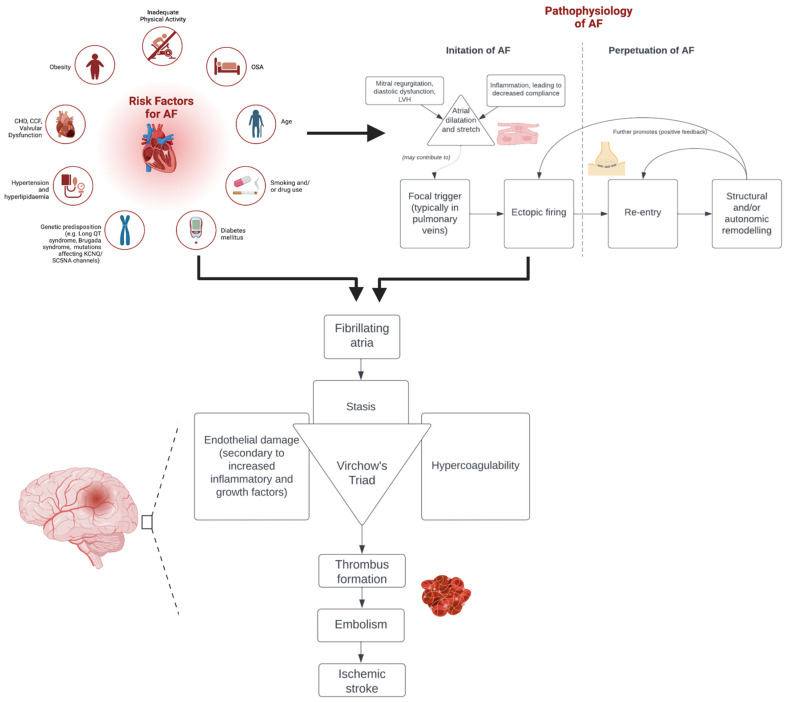
Risk factors and pathophysiology of atrial fibrillation. Abbreviations: AF = atrial fibrillation, CHD = coronary heart disease, CCF = congestive cardiac failure, KCNQ = potassium channel voltage-gated Q subfamily, SC5NA = sodium voltage-gated channel type 5 subunit alpha, OSA = obstructive sleep apnoea, and LVH = left ventricular hypertrophy.

Atrial fibrillation (AF) is the most prevalent cardiac arrhythmia worldwide [1] and is a major cause of morbidity [2] and mortality [3]. It has a global prevalence of approximately 0.51%, increasing to 10–17% in those over age 80 [4]. AF is characterised by ectopic depolarisations, which lead to asynchronous atrial contractions and irregular ventricular activity [5]. Patients may report palpitations, dyspnoea, lethargy, dizziness, and chest pain [6] but are often asymptomatic [7]. AF has various subtypes, including paroxysmal, persistent, and permanent AF [8]. Whilst multiple cardiovascular conditions such as coronary heart disease and hypertension may influence or exacerbate AF [7], a primary clinical consideration is the association of AF with a high risk of acute ischemic stroke (AIS). The management of AF in the setting of AIS is a challenging clinical scenario. Stasis due to fibrillating atria predisposes to cardiac thrombus formation by Virchow’s Triad [9], most prominently in the left atrial appendage (Figure 1). These thrombi may embolise the cerebral circulation and cause AIS or transient ischemic attacks (TIAs) [10]. Approximately 23.7% of AIS or TIA patients have underlying AF; however, AF is often undiagnosed due to insufficient cardiac monitoring [11]. Even after adjusting for other factors, AF patients remain at a three-to-five-fold increased risk of acute stroke [9]. They are additionally more likely to experience poor functional outcomes and have a higher rate of mortality following stroke [12]. This is even more poignant in the era of reperfusion therapy, such as intravenous thrombolysis (IVT) and endovascular thrombectomy (EVT), which is currently the cornerstone of AIS management [13]. However, the outcomes after reperfusion therapy are not consistent across all stroke subgroups, with AF being an important factor mediating this. This article provides an overview of the diagnosis and management of AF in the setting of AIS, as well as the prevalence and outcomes of AF patients in AIS patients receiving reperfusion therapy.

Whilst the cause of AF is often undetermined, there are several factors that elevate the risk of developing arrhythmia. Age is the most significant risk factor, but a range of cardiovascular conditions also increase the risk of developing AF [14]. For example, the increased afterload in hypertension is linked to left ventricular hypertrophy and consequent diastolic dysfunction and left atrial enlargement. All these factors increase the stretch on the left atrium, which has been demonstrated to increase the risk of AF [15]. Eventually, there is a risk of ectopic focal firing leading to a paroxysmal AF, of which 90% is associated with the pulmonary veins [16]. However, AF may become persistent when this progresses to larger re-entry circuits. Eventually, irreversible electrical and structural remodelling may cause permanent AF. This remodelling leads to further ectopic foci and re-entry events, thus leading to a positive feedback loop [16]. However, this progression from paroxysmal to persistent and permanent AF is not inevitable, and there are multiple instances of persistent AF spontaneously becoming paroxysmal [14].

## 2. Link between Atrial Fibrillation and Acute Ischemic Stroke

There are multiple pathophysiological reasons for the link between AF and AIS. The dysrhythmic atria in AF may lead to stasis of blood, which is most prominent in the left atrial appendage [17]. Whilst this is the main factor contributing to thrombosis, AF is also associated with biomarkers of inflammation and platelet activation that may lead to endothelial damage and hypercoagulability [16]. Ultimately, thrombi may form via Virchow’s triad and subsequently embolize the cerebral circulation, causing AIS. Furthermore, AF is associated with increased age and higher rates of hypertension, ischemic heart disease, diabetes mellitus, and heart failure, all of which are factors that increase the risk of stroke [9].

## 3. Diagnostic and Therapeutic Management of Atrial Fibrillation

An overview of the current diagnosis and management of AF in the setting of AIS based on the current evidence-base and recommendations is discussed below.

### 3.1. Diagnosis of Atrial Fibrillation

**Figure 2:**
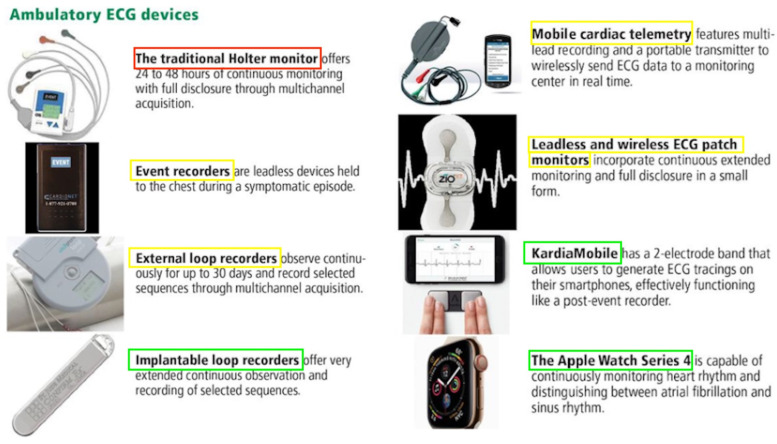
Devices used for ambulatory electrocardiogram monitoring. Red = devices utilised for early secondary stroke prevention via arrhythmia detection, yellow = devices which may be used for secondary stroke prevention in the first 30 days, and green = devices that specialise in long-term secondary stroke prevention over 30 days. Adapted from Sanders et al. Cleveland Clinical Journal of Medicine (2019) [38]. Abbreviations: ECG = electrocardiogram.

Considering the association between AF and AIS [18], appropriate diagnosis of AF and subsequent treatment is vital to prevent stroke [19]. The diagnosis of AF in the setting of AIS is usually based on electrocardiographic (ECG) findings. However, AF may be paroxysmal and not detectable on a single ECG [20]. Therefore, prolonged ECG monitoring, such as telemetry or Holter monitoring, may be required to establish the diagnosis of AF [21]. Transthoracic or transoesophageal echocardiography may be useful in identifying underlying structural heart disease, such as left atrial enlargement or valvular abnormalities [22]. Unfortunately, there are limited organised screening procedures employed across the general population [23,24]. In patients with suspected AF, a range of ambulatory cardiac monitoring devices may be utilised [25] (Figure 2). In-hospital and post-discharge cardiac monitoring is also commonly undertaken following stroke, as detecting AF can help determine the cause of stroke [26] and guide treatment to prevent stroke recurrence [27]. Following AIS, the identification of AF plays a crucial role in enhancing secondary prevention strategies. A study by Boriani et al. compared AF detection and oral anticoagulant (OAC) initiation in ischemic stroke patients using insertable cardiac monitors (ICMs) versus external cardiac monitors (ECMs) [28]. Over 24 months, 33.9% of ICM-monitored patients were diagnosed with new AF compared to 13.3% with ECMs. Consequently, more ICM patients received OAC prescriptions (35.9% vs. 16.8% for ECM patients). Put simply, ICM-monitored ischemic stroke patients were nearly three times more likely to be diagnosed with AF and prescribed OACs. However, this heightened detection and intervention did not translate to a reduction in stroke risk or mortality. Consistent with prior studies [29,30,31], this study highlighted that the use of ICMs may improve AF detection rates and OAC prescription among AIS patients. Nevertheless, it also brings to attention the clinical implications of improved AF detection in an older, medically complex population. Whilst the improvements in mortality or stroke risk were not observed in this study, previous studies have revealed that improved AF detection may lead to better treatment and, hence, reduction in risk of stroke or death [27,31]. A meta-analysis by Tsivgoulis et al. [32] revealed that ICM use, as compared to conventional monitoring in cryptogenic stroke, was associated with improvements in AF detection, increased initiation of OAC, and subsequent lower risks of recurrent stroke. Continuous monitoring for AF proves especially clinically significant in cases where AF occurrences are infrequent, paroxysmal, and asymptomatic [33,34]. This is particularly relevant for patients with cryptogenic stroke, as detecting AF and administering OACs can have a notable impact due to the influence of silent brain infarcts on cognitive function in AF patients [35]. Alongside the diagnostic challenges of AF detection, other factors may also play a role, including peri-hospital systems and workflows [36,37].

In addition to the inpatient ECGs that are routinely conducted for patients admitted with palpitations, syncope, or following a stroke, there are multiple devices that may be used to detect or monitor arrhythmias in a real-world scenario [38]. Figure 2 depicts some of the common devices that are used and outlines their basic mechanisms. Furthermore, continuous rhythm monitoring allows for a better understanding of various AF patterns over time, shedding light on the progressive remodelling of the atrial substrate or the deterioration of underlying diseases. The Fitbit Heart Study, encompassing 455,699 participants, found that wearable devices leveraging optical photoplethysmography (PPG) sensors and software algorithms demonstrated a high positive predictive value for detecting AF concurrently and in identifying individuals likely to have AF during subsequent ECG patch monitoring [39]. This suggests that wearable devices have the potential to assist in identifying individuals with undiagnosed AF. However, there are uncertainties in the literature regarding the optimal approach to AF detection, and these are exacerbated by rapidly developing technologies [40,41] and a lack of standardised protocols between different centres [24]. It is thus essential for clinicians to be aware of the current literature that compares the timescales and detection rates of these monitoring devices (Table 1)**.**

### 3.2. Treatment of Atrial Fibrillation

The management of AF in the setting of AIS should be guided by the underlying aetiology of the stroke, the severity of the stroke, and the risk of recurrent stroke. The primary goals of management are to prevent recurrent stroke [37] and to maintain cardiac hemodynamic stability [21].

*Anticoagulation:* Anticoagulation is the cornerstone of stroke prevention in patients with AF. The decision to initiate anticoagulation should be based on the risk of stroke and bleeding [49]. The CHA2DS2-VASc score is commonly used to assess stroke risk in patients with AF [25]. For patients with a CHA2DS2-VASc score of 2 or more, anticoagulation with a vitamin K antagonist (VKA) or a direct-acting oral anticoagulant (DOAC) is recommended [25]. VKAs such as Warfarin are indicated in patients with valvular AF, which refers to those with moderate-to- severe mitral stenosis or mechanical heart valves [49]. However, most AF patients are recommended DOACs such as dabigatran, apixaban, rivaroxaban, and edoxaban. The shift in guidelines of recommending DOACs in most AF patients was prompted by seminal clinical trials comparing warfarin with the four aforementioned DOACs, respectively [50,51,52,53]. A recent real-world study from Japan demonstrated that DOACs exhibit notably superior safety and effectiveness profiles when compared to warfarin in elderly patients diagnosed with nonvalvular AF [54]. The current literature reinforces this shift in anticoagulant choice, with recent meta-analyses demonstrating superior efficacy and safety with DOACs (Table 2). In cases of ischemic stroke with AF, anticoagulation therapy is preferred over antiplatelet therapy for thromboprophylaxis [55]. However, for ischemic strokes unrelated to AF, anticoagulant therapy does not show incremental benefits and may carry higher risks compared to antiplatelet therapy [56]. The main concern with antithrombotic therapy is the risk of bleeding, especially when combining antiplatelets and anticoagulants, particularly in patients with bleeding history or other risk factors. The question arises about combining these therapies for ischemic stroke patients with AF and large-artery atherosclerosis. A study by Kim et al. [57] in South Korea found no additional benefit and a greater risk associated with combining antithrombotic and antiplatelet therapy in such patients, indicating OAC monotherapy as an optimal antithrombotic regimen in preventing recurrent stroke in ischemic stroke due to AF and large-vessel atherosclerosis. However, the study had limitations, including the inclusion of individuals treated with VKAs, known for higher bleeding risk compared to direct oral anticoagulants. Another observational study examined the impact of antithrombotic medications in patients with both AF and cerebral microbleeds [58]. It found that combining antiplatelets and anticoagulants can be detrimental in patients with cerebral microbleeds and AF, confirming previous research that such combination therapy does not provide added protection against ischemic stroke in AF patients. Additional research is necessary to optimise stroke prevention strategies for at-risk populations such as those with AF and cerebral microbleeds. Future trials are needed to explore novel combinations of antiplatelets with DOACs or other drug classes (e.g., factor XII antagonists) to determine their effectiveness and safety.

However, continuing anticoagulation in the acute setting of ischemic stroke is controversial, as it may increase the risk of haemorrhagic transformation (HT) [63]. The American Heart Association/American Stroke Association (AHA/ASA) recommends withholding anticoagulation for at least 24 h after IVT or EVT in patients with AF-related AIS, with early initiation of anticoagulation reserved for high-risk patients [13]. However, recent randomised controlled trials (RCTs) suggest that early initiation of DOACs may be safe and effective in selected patients with AF-related AIS [64,65].

AF known before an ischemic stroke (KAF) is postulated to be an independent category with a higher recurrence risk compared to AF detected after stroke (AFDAS) [66,67,68,69,70,71]. It raises the possibility that the difference in risk may be influenced by pre-existing anticoagulation, which is more common in KAF and signifies a heightened risk of ischemic stroke recurrence [72]. A recent study by Lyrer et al. [73] challenged the idea that KAF and AFDAS are distinct prognostic entities and suggest that, rather than solely attributing a high stroke recurrence risk to KAF, future research should concentrate on understanding the causes of stroke in patients despite anticoagulation treatment to develop more effective preventive measures.

**Figure 3:**
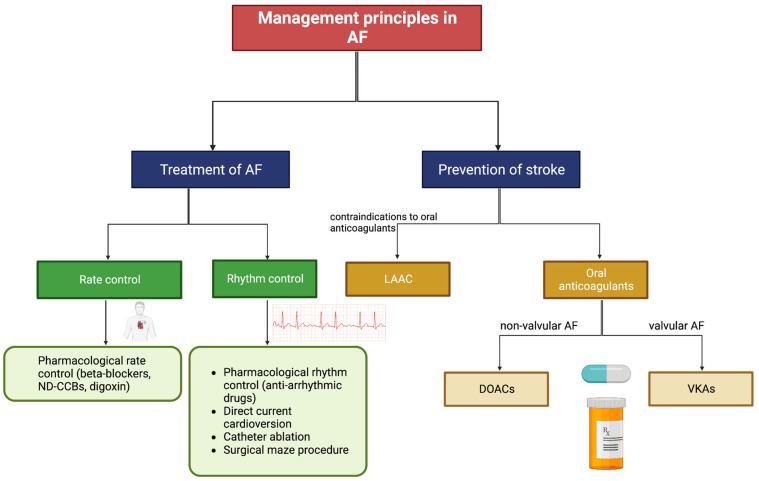
Patient management options for AF. Abbreviations: AF = atrial fibrillation, ND-CCBs = non-dihydropyridine calcium channel blockers, LAAC = left atrial appendage closure, DOACs = direct-acting oral anticoagulants, VKAs = vitamin K antagonists. Adapted from Li et al. (2020) Cardiovascular Journal of Africa [74].

Alongside stroke prevention and the management of AF risk factors, there are various methods of treating AF directly (Figure 3). These treatments are broadly categorised into the aims of restoring sinus rhythm (rhythm control) or controlling the heart rate (rate control) [74]. Despite the wide range of options, a large portion of AF patients remains undertreated [75]. Both undertreatment and underdiagnosis are particularly common in developing nations [76]. These regions are further plagued by a lack of local studies investigating the epidemiology and clinical outcomes in AF patients experiencing AIS.

AF is linked to cognitive decline and various forms of dementia, often attributed to brain injuries resulting from macro- and microembolic events [77,78]. Emerging evidence supports the strategic utilisation of anticoagulation in AF patients, including the timing and appropriate administration, to specifically reduce the likelihood of dementia [79,80,81]. In a large observational cohort study involving over 142,000 patients aged 50 and older with nonvalvular atrial fibrillation (AF), the use of oral anticoagulants was linked to a decreased risk of dementia, especially in patients aged 75 or older (Hazard Ratio, 0.84) [82]. These findings emphasize the benefits of anticoagulation in reducing dementia risk, even in the absence of a significant prior stroke. This study reinforces the importance of early AF diagnosis, ongoing anticoagulant use, and its potential role in lowering the risks of stroke, cognitive decline, and dementia.

Managing cardiovascular, metabolic, and lifestyle risk factors, as well as upstream therapies that indirectly target the mechanisms of AF, aids in the prevention of AF [74]. When AF is diagnosed, there are various options to achieve rhythm control (restoring sinus rhythm) and/or rate control (slowing the heart rate). Pharmacological rhythm control is useful in preventing AF progression and harmful remodelling, whilst rate control reduces hospitalisation but is less effective at resolving the arrhythmia itself [83]. Catheter ablation is a common procedure used to treat AF and is reported to have a higher success rate than pharmacological therapy [84,85,86,87]. The invasive nature of ablation carries risks, but the overall rate of adverse events is comparable to that of pharmacological treatments [85,86]. AF patients are commonly prescribed oral anticoagulants, which prevent stroke by restraining thrombosis [59]. Patients who have contraindications to oral anticoagulants may undergo LAAC, which prevents stroke by blocking thrombi from exiting the left atrial appendage [88].

*Early* vs. *later anticoagulation:* When to initiate anticoagulation for long-term secondary stroke prevention, especially in individuals with AF, is a critical question [89]. It revolves around determining the point at which the risk of haemorrhagic complications resulting from anticoagulation is balanced by the positive impact it has in preventing recurrent strokes. Hospital-based cohort studies have indicated the feasibility of early DOAC initiation within 1, 2, 3, or 4 days according to stroke severity in reducing the risk of recurrent stroke or systemic embolism without an increase in major bleeding [90]. In a recent international open-label trial involving 2013 patients with AF who had experienced an AIS, the introduction of DOACs within 2 days after a minor/moderate stroke and 6–7 days after a major stroke, as determined by imaging assessments, resulted in a similar risk for a combined safety and efficacy outcome at both 30 days (Odds ratio [OR], 0.57) and 90 days (OR, 0.60) [91]. These findings indicate that the early administration of systemic anticoagulation with a DOAC following an AIS is safe when guided by severity classification based on imaging.

*Rhythm control:* To control the irregular rhythm in AF, treatment strategies such as antiarrhythmic drugs and cardioversion may be employed [87,92]. A meta-analysis of 447,202 AF patients demonstrated that early rhythm control was associated with significant reductions in the risk of stroke or systemic embolism [93]. Compared to patients receiving only pharmacological treatments, those treated with catheter ablation have reported lower rates of stroke and mortality [87]. Recent studies suggest that early rhythm control is also safe and effective in selected patients with AF-related AIS [94,95,96].

*Rate control:* Treatments to slow the heart rate in AF patients, such as beta-blockers, non-dihydropyridine calcium channel blockers, and digoxin, provide symptom relief whilst reducing the risk of thromboembolic complications [97]. Despite their inability to restore sinus rhythm, a meta-analysis revealed no significant differences in the odds of stroke and all-cause mortality in patients receiving medications to achieve rate control compared to pharmacological rhythm control [98].

## 4. Impact of Atrial Fibrillation on Stroke Outcomes

Alongside being an established risk factor for stroke, AF is a predictor of poor outcomes following AIS [99]. Outcomes measures include the modified Rankin Scale (mRS) [100], where a score of zero to two typically represents favourable functional outcomes, and three to six typically reflects poor functional outcomes. Other outcomes include HT, symptomatic intracerebral haemorrhage (sICH), and mortality. A prospective study on 10,528 AIS patients revealed that AF was associated with an increased risk of mortality and severe disability [101]. However, multivariate analysis revealed that this association was mostly due to the increased age and greater initial stroke severity in AF patients. A recent study investigating long-term prognosis in AIS patients with and without AF demonstrated a significantly higher rate of one-year mortality post-stroke in AF patients [102]. Similarly, this association was no longer significant after adjusting for comorbidities such as heart failure and the greater age and baseline stroke severity in AF patients. Nevertheless, the unfavourable prognosis of AF patients remains of considerable clinical importance, and potential reasons for this association have been studied extensively [103]. The poorer outcomes observed in AF patients have been linked to greater volumes of hypoperfusion at baseline following AIS and the higher frequency of HT in AF patients [99]. Adequate oral anticoagulation is paramount in preventing the poor prognosis of AF patients. Compliance with oral anticoagulants in AF patients reduces the risk of stroke and is associated with lower mortality and disability following AIS [104]. Oral anticoagulants also reduce the risk of recurrent stroke in AF patients, which is a substantial source of morbidity and mortality in the setting of AF [105]. Furthermore, there is increasing awareness that poorer collateral perfusion is a predictor that mediates suboptimal clinical outcomes in AIS patients with AF [106,107,108]. A retrospective study revealed that AF was significantly more likely to be present in AIS patients with poor collateral status compared to those with good collateral status [107]. A 2023 study demonstrated that in patients with poor collateral flow, a favourable functional outcome was found in 26.7% of AF patients compared to 51.2% of non-AF patients [106]. In patients with good or moderate collateral statuses, there was no significant difference in the rate of favourable functional outcomes between AF and non-AF patients. Therefore, interventions to improve collateral perfusion in AF patients are vital for improving prognosis. One such intervention is premorbid treatment with statins, which has been linked to better or excellent collaterals in AF patients experiencing stroke [109].

## 5. Reperfusion Therapy in Acute Ischemic Stroke

AIS is a medical emergency that requires prompt management to prevent permanent neurological damage [13]. Reperfusion therapy, such as IVT or EVT, is the mainstay of treatment for patients with AIS who are eligible for these interventions [13]. Whilst the use of reperfusion therapy has become the standard of care in clinical practice, it remains unclear whether the presence of AF modulates its effectiveness [110,111]. Resolving this uncertainty is crucial for formulating ideal treatment strategies as well as for risk stratification and communicating prognosis with patients [12]. The next section of this article discusses current studies regarding the outcomes after reperfusion therapy in AF patients with AIS and subsequently critically analyses the strengths and limitations of this literature.

### 5.1. Impact of Atrial Fibrillation on Outcomes after Intravenous Thrombolysis

IVT is a time-sensitive management strategy that aims to restore blood flow following AIS by cleaving the fibrin network of clots and causing them to dissolve [112]. It may be contraindicated in AF patients taking warfarin who have an international normalised ratio (INR) greater than 1.7 and patients taking DOACs within the past 48 h who also have abnormal coagulation markers [13]. However, AF itself is not a contraindication for IVT [13]. Several studies have demonstrated that AF is associated with lower odds of favourable functional outcomes following IVT compared to non-AF patients [113,114,115,116]. A large registry-based study of 7193 patients further demonstrated that AF was a predictor of sICH following IVT [117]. These findings are reinforced by meta-analyses which found that AF was associated with significantly lower odds of favourable functional outcomes as well as a higher risk of sICH and mortality after IVT [110,118]. However, some cohort studies have found no significant associations between AF and functional outcomes, sICH or mortality following IVT [119,120]. Intriguingly, one of these studies suggested that AF was linked with favourable outcomes in the subgroup of patients with high initial stroke severity [120]. Furthermore, a prospective study revealed that the association between AF and poorer functional outcomes following IVT was no longer present after adjusting for confounders such as age, National Institutes of Health Stroke Scale (NIHSS) score, and comorbidity status [121]. Consequently, it is imperative to re-evaluate the current literature to understand how AF influences the outcomes following IVT. The findings from existing meta-analyses on this topic are summarised in Table 3.

### 5.2. Impact of Atrial Fibrillation on Outcomes after Endovascular Thrombectomy

Cardiac emboli in AF patients are more likely to result in large vessel occlusion strokes affecting the anterior cerebral circulation [123]. EVT is a technique that may be utilised to mechanically retrieve these clots [13]. Several studies have suggested there is no significant association between AF and the rates of favourable functional outcomes, sICH, and mortality following EVT [124,125,126,127]. However, a 2021 prospective study of 245 patients revealed that AF patients receiving EVT had a significantly higher rate of ICH [128]. A recent prospective study of 127 patients also found that AF was associated with significantly lower odds of favourable functional outcomes and higher odds of mortality following EVT [129]. Contrastingly, after adjusting for other variables, a single-centre study demonstrated that AF was associated with improved functional outcomes following EVT, with no difference in the rates of sICH and mortality [123]. When these studies were compiled into the meta-analysis by Kobeissi et al. [130], the rates of favourable functional outcomes and sICH were similar, though AF patients receiving EVT had a significantly higher rate of mortality. With the precise impact of AF on outcomes following EVT remaining undetermined, this is an area of substantial clinical and research interest [130]. Table 4 summarises the outcomes from meta-analyses investigating the relationship between AF and outcomes following EVT.

Bridging therapy (BT) refers to the administration of IVT followed by EVT [131]. It is currently recommended in patients eligible for both IVT and EVT [13]. Whilst AF is not a contraindication to BT, there are suggestions that AF patients may be less likely to benefit from this treatment approach compared to non-AF patients [132]. An international cohort study revealed that providing AF patients with IVT prior to EVT was associated with an increased risk of sICH without improving functional outcomes [132]. This effect was not observed in patients without AF. Similarly, a multicentre study found that BT led to a significantly higher proportion of favourable functional outcomes compared to EVT alone in patients without AF but not in patients with AF [131]. However, after adjusting for confounders, a 2022 study reported that there was no significant association between AF and the likelihood of favourable functional outcomes, sICH, or mortality following BT [133]. Despite this being an area of growing interest [132], there are currently limited studies investigating the relationship between AF and outcomes following BT. The findings from these studies are described in greater detail in Table 5.

## 6. Discussion

AF is implicated in a sizeable portion of ischemic strokes [11], and these patients are vulnerable to significant functional impairment and mortality despite receiving treatment [110]. Although AF is an established risk factor for stroke [134], there is limited research on the prevalence of AF amongst AIS patients receiving reperfusion therapy. Variations in AF prevalence and underdiagnosis may contribute to inconsistencies in the outcomes following reperfusion therapy [20]. Analysing these variations can help guide workflows and systems planning to better tailor reperfusion therapy in high-risk cohorts such as AF patients. It may also reveal regions where a particular form of reperfusion therapy is being overused or withheld among AF patients, as well as regions where AF is more likely to be underdiagnosed [135]. Identifying these areas of need is vital in allocating funding and resources for more comprehensive cardiac monitoring [136]. The varying AF prevalence within cohorts receiving IVT, EVT, and BT is detailed in Table 3, Table 4 and Table 5, respectively. To the best of our understanding, our meta-analysis is the first study that reports on the pooled prevalence of AF in AIS patients receiving these treatments [122]. Our findings demonstrate a pooled AF prevalence of 31%, 42%, and 36% in patients treated with IVT, EVT, and BT, respectively. These findings reinforce the substantial contribution of AF in the realm of AIS and highlight that the burden of AF is particularly prominent in the context of EVT. Furthermore, the study provided insight into regional variations in AF prevalence, such as a higher prevalence in East Asian nations and instances of lower AF prevalence in developing nations where there is limited access to technologies for diagnosing AF [122].

AF is more common among individuals with heart conditions. A recent study found that congestive heart failure (CHF) and left atrial enlargement (LAE) are strong indicators of AF in stroke patients, particularly in cases of ischemic stroke caused by large- or small-vessel disease [137]. This suggests that using ICMs may be particularly beneficial for these patients as part of secondary stroke prevention. While implantable loop recorders have improved AF detection in non-cardioembolic stroke patients compared to standard care, it is important to note that the long-term benefits of widespread AF screening using these devices for all stroke patients have not been definitively established [138]. In addition to the diagnostic challenges of AF detection, other factors may also play a role, including peri-hospital systems and workflows.

Beyond stroke, another subgroup of patients at high risk of stroke are epilepsy patients with AF. This is poignant as post-stroke seizures and post-stroke epilepsy are common in emergency settings [139]. A study of 96 epilepsy patients, 44 AF patients, and 77 individuals without cardiovascular or neurological conditions revealed that the variability in *p*-wave morphology, a marker of atrial tissue activation/conduction irregularities, was greater in the epilepsy group than in the control group and neared levels observed in AF patients [140]. Though further confirmation in larger epilepsy patient cohorts may be needed, these initial results suggest that individuals with chronic epilepsy may undergo accelerated structural changes and cardiac electrical instability. This supports the concept of an “epileptic heart” condition and suggests that using specific electrocardiographic traits to identify at-risk patients could be beneficial. This may also have implications for post-stroke epilepsy.

The impact of AF in determining the outcomes following reperfusion therapy is also controversial. Consistent with previous studies [110,118], our meta-analysis found that AF was associated with significantly lower odds of favourable functional outcomes and greater odds of sICH and 90-day mortality in patients receiving IVT [122]. However, there were no significant associations between AF and these clinical outcomes in patients receiving EVT. A limitation of existing meta-analyses is that they often do not adjust for comorbidities and the baseline characteristics of patients [130]. AF patients are commonly older [99,111,129] and likely to present with more severe strokes on admission [123,129]. Both factors have been linked to poorer outcomes [129,141,142,143]. Conversely, a meta-analysis of six RCTs was able to adjust for covariates, eventually reporting similar outcomes among AF and non-AF patients receiving EVT [127]. Future studies that account for these factors are essential to enable more valid conclusions to be drawn.

There are various hypotheses regarding why AF may influence the outcomes following reperfusion therapy in AIS patients. Since cardioembolic thrombi, such as those in AF, are predominantly formed secondary to stasis in cardiac chambers [103], it was hypothesised that this might cause them to contain a greater proportion of erythrocytes relative to fibrin [144]. It was further theorised that the thrombi in AF patients might be more resistant to IVT, as IVT targets fibrin rather than erythrocytes [103]. These hypotheses have been challenged recently, with a 2022 meta-analysis [145] revealing that cardiac emboli had a significantly higher fibrin content. It also highlighted that IVT was conversely more successful in lysing clots with greater proportions of erythrocytes, potentially due to the less developed fibrin networks. Further histopathological investigations are necessary, particularly those that exclude prior IVT, as this may alter clot composition [146].

Patients with AF may be likely to have strokes with emboli that are larger and more resistant to EVT [125]. Despite this, a 2023 meta-analysis revealed that successful recanalisation rates were similar among AF and non-AF patients [130]. Comparatively more widespread intracerebral atherosclerosis in non-AF patients experiencing AIS [147] may worsen prognosis in this group [123] and explain why the outcomes are often similar overall. However, patients with greater cerebral atherosclerosis are also likely to develop a more comprehensive collateral circulation in response to chronic hypoperfusion [148]. This contrasts with AF patients, where poorer collateral circulation is a factor that worsens prognosis following AIS [110]. Considering these concurrent mechanisms, further research is required to understand if there is a pathophysiological basis for AF patients to respond more poorly or favourably to EVT.

Additionally, there are currently no meta-analyses and markedly fewer independent studies that evaluate the influence of AF on outcomes following BT compared to those analysing IVT or EVT alone. The existing literature suggests that AF patients often respond poorly to BT relative to their non-AF counterparts [131,132] (Table 5). It is hypothesised that the additional IVT may elevate sICH risk without improving functional outcomes [132]. There are thus suggestions that AF patients who are eligible for both treatments may benefit from solely receiving EVT [125]. However, there are currently not sufficient studies to conduct a robust meta-analysis on this topic. Consequently, more data is required before conclusions can be drawn and applied to clinical practice.

### 6.1. Limitations

Study design: Most studies in this field are of retrospective design, which are subject to selection bias [123] as well as missing data and consequent recall bias [149]. Whilst previous IVT meta-analyses have included subgroup analyses to examine how results varied between prospective and retrospective studies [110,118], such deconstructions were absent within previous EVT meta-analyses [130]. This limits their effectiveness as it may conceal biases that disproportionately affect a particular study design. The meta-analysis from our team accounted for this by providing forest plots that compare the results between prospective and retrospective studies [122]. RCTs are markedly less common in this field, which may partly be attributed to difficulties in obtaining informed consent alongside the duty to provide the best possible care [150]. Nevertheless, the meta-analysis by Smaal et al. [127] on outcomes following EVT contained six RCTs. Unfortunately, AF was not a central focus of these RCTs. There was thus less detailed reporting regarding the effect of AF on outcomes, and limited characterisation of the AF subtypes within each study population [127,151].

Sample size: The validity of several studies is undermined by their relatively small sample sizes. For example, a prospective investigation by Padjen et al. [152] reported considerably greater odds of favourable outcomes in AF patients treated with IVT compared to AF patients not treated with IVT. However, the small sample size of only 34 AF patients receiving IVT may explain the vastly different results relative to a meta-analysis comparing the same groups [110]. Furthermore, in that same study, sICH was reported in only two and four IVT-treated and untreated AF patients, respectively [152]. Multiple other investigations similarly reported single-digit incidences of sICH in the AF and/or non-AF groups [75,121,123,128,129,153]. This increases the susceptibility to sampling error, introducing biases that may undermine the results of both independent studies and smaller meta-analyses [154].

Method of reporting functional outcomes: A strength of the existing literature is that most studies are consistent in using an mRS score of zero to two to represent favourable 90-day functional outcomes, with a score of three-to-six representing poor or unfavourable outcomes. This facilitates easier comparisons between studies. However, the dichotomisation of favourable vs. poor outcomes may conceal differences in outcomes at specific segments along the spectrum of mRS scores [121,123]. The studies that depict the entire distribution of mRS scores for each patient group [123,142,152,153,155] are often able to accentuate these details. For example, Fu et al. [123] highlighted that whilst AF patients were more likely to experience a complete recovery (15.8% vs. 10.1%), they also more commonly had a severe mRS of five to six (29.8% vs. 23.6%).

Patient variations related to atrial fibrillation: AF is a highly heterogeneous condition [156], and there are sizeable variations in how studies account for patient-to-patient variations regarding the method of AF detection, AF subtype, and the cause of stroke.

Atrial fibrillation detection: Several studies do not describe the method of AF detection and presumably drew from databases or patient records to identify if patients had pre-existing AF [103,111,123,125,131]. Where a detection method is described, the most common approach involves analysing existing patient records alongside a 12-lead ECG on hospital admission, followed by 24–72 h of continued cardiac monitoring [121,133,151,152,153]. However, the exact protocols differ at each centre. Protocols vary to the extent that one study used solely a 12-lead ECG on admission for those without previously diagnosed AF [155], whilst another employed continual in-hospital monitoring and at least 30 days of cardiac monitoring post-discharge [75]. These variations compromise the accuracy and reliability of the results since less stringent detection methods typically lead to underestimations of AF prevalence [20,42,47,48].

Subtype of atrial fibrillation: An additional benefit of extended cardiac monitoring is that it may help determine the prevalence of different AF subtypes [157]. Despite some studies effectively describing the prevalence of each subtype, patients with these heterogeneous subtypes of AF patients were often combined in the final analyses [121,133,152]. One of these studies [121] justified this using findings from Seet et al. [153], who reported non-significant differences in the outcomes following IVT between patients with paroxysmal AF compared to those with persistent or permanent AF. However, this investigation had a small sample of only 21 patients with paroxysmal AF, exposing it to type II error [153]. Even if AIS risk is assumed to be similar among paroxysmal and persistent AF patients [158], this only reinforces the importance of more comprehensive cardiac monitoring. This is because the transient nature of paroxysmal AF makes it more difficult to diagnose by short-term monitoring [20,38], and miscategorising these patients would be more detrimental if they do indeed have analogous outcomes to other AF patients.

Cause of stroke: Not all strokes in AF patients arise from cardiac emboli, but 40–50% of cases arise due to intracranial thrombi or emboli from other sources [118]. This is exacerbated by the higher rates of comorbid cardiovascular risk factors in AF patients [159,160]. However, the nature of the clot and the cause of the stroke usually remain unknown since the stroke current workup usually does not include histopathology [161].

### 6.2. Policy Recommendations

This literature review has revealed multiple pain points in the field of AIS in AF patients. The recommendations for future policy and research are:

1. Conducting an RCT that investigates how AF influences outcomes after reperfusion therapy since current data is primarily based on cohort studies. For RCTs investigating other hypotheses in AIS patients receiving reperfusion therapy, we strongly recommend that effort is made to ensure a standardised protocol for diagnosing AF and that the prevalence of different AF subtypes is reported where possible;
2. Further trials that investigate how AF influences outcomes following BT. Despite being an increasingly contested field, the current lack of data prevents concrete conclusions from being drawn;
3. Greater implementation of technologies for the detection of AF following AIS. Given that the prevalence of AF is highest in EVT cohorts, this may justify a greater allocation of resources toward these centres;
4. Conduct cost-effectiveness analyses across a range of cardiac monitoring devices, including mobile cardiac outpatient telemetry (MCOT), implantable loop recorders, and wearable devices. Use this to guide policy on the most suitable protocol for diagnosing AF following AIS. In particular, research the efficacy of utilising MCOT for the first 30 days post-stroke followed by long-term monitoring with implantable loop recorders in patients with ischemic stroke of undetermined origin;
5. Whilst long-term cardiac monitoring has been shown to be effective in AF detection, further research is required to investigate the clinical benefits of widespread AF screening;
6. Further studies on the prevalence of AF in developing and under-resourced parts of the world. There is currently an alarming deficiency of AF-related research in these regions;
7. Increase the awareness of AF among healthcare workers and the general population. Provide education programs among primary healthcare workers for the early detection of AF. Even interventions as simple as routine 30 s pulse checks and opportunistic ECG recordings may be highly useful without significant cost. To facilitate primary prevention of AF, provide funding for community-based activities that raise awareness about AF, and link AF with stroke awareness campaigns.

## 7. Conclusions

AF is a significant risk factor for AIS and is often associated with poorer prognosis following reperfusion therapy. Our understanding of how AF mediates outcomes following reperfusion therapy is evolving. Despite several investigations on the role of AF in reperfusion therapy, most studies are retrospective and have inconsistent approaches to AF detection as well as limited reporting of the different AF subtypes. There is also a need for future studies that adjust for covariate factors and account for differing study designs. Albeit AF was associated with a worse prognosis in contrast to the non-AF population, benefits of reperfusion therapy, IVT, and/or EVT prevail in AIS patients with or without AF. Further analyses on the prevalence of AF in AIS patients and the effect of AF on outcomes after reperfusion therapy may provide additional insights and inform guidelines to optimally manage AIS patients with AF.

## Figures and Tables

**Table 1:** Studies comparing the detection rates of atrial fibrillation between different cardiac monitoring techniques.

Study	Number of Patients (n)	Study Design	Technique(s) and Timescale	Outcomes (Primarily Regarding AF Detection Rate)
Buck et al. [42]	300	RCT	12-month ILR vs. 30-day ELR	In ischemic stroke patients monitored for 12 months using an ILR, AF was detected in 15.3% of patients compared to 4.7% of patients monitored with a 30-day ELR (RR 3.29 [95% CI 1.45–7.42], *p* = 0.03).
Koh et al. [43]	203	RCT	30 days of KardiaMobile ECG (used for 30 s 3 times per day) vs. one additional round of 24-h Holter monitoring	In patients with a recent cryptogenic stroke or TIA, AF of duration ≥ 30 s was detected in 9.5% of patients in the KardiaMobile group compared to 2.0% in those receiving 24 h Holter monitoring (*p* = 0.024).
Liu et al. [21]	158	Prospective	14-day ECG patch vs. 24-h Holter monitoring	AF and/or atrial flutter was detected in 9.5% of patients wearing the 14-day ECG patch compared to 3.8% in patients with 24 h Holter monitoring (*p* = 0.042).
Medic et al. [44]	1000	Retrospective (economic model)	30-day MCOT followed by ILR vs. ILR alone over 12 months	In patients with cryptogenic stroke, 30-day MCOT followed by an ILR had an AF detection rate of 20.9% compared to a detection rate of 4.5% when using an ILR alone. Cost-effectiveness analysis revealed 7.72 times lower costs per AF patient detected when using MCOT initially (USD 29,598 per patient with detected AF) compared to when using an ILR only (USD 228,507 per patient with detected AF).
Chua et al. [20]	32	Prospective	14-day ECG patch vs. 24-h Holter monitoring	Paroxysmal AF and/or atrial flutter was detected in 19% of patients wearing the ECG patch compared to 3% in patients with 24 h Holter monitoring (*p* < 0.05).
Perez et al. [45]	419,297	Prospective	8 months of monitoring with an Apple Watch	Of Apple Watch wearers among the general population, 0.52% received a notification regarding an irregular pulse over 8 months. Of the patients who were notified to have an irregular pulse and subsequently wore and returned an ECG patch, AF was confirmed in 34%. Comparatively, in those who did not receive a notification regarding an irregular pulse, a diagnosis of AF was established in 1.0% of patients.
Derkac et al. [46]	78,490	Retrospective	MCOT vs. ILR analysed over 8 months	AF was diagnosed in 23.5% of patients with MCOT compared to 11.3% of patients with an ILR. It should be noted that the median prescription time for the MCOT group was 20 days compared to 30 days in those with the ILR group, despite the latter device having the potential to be used for a considerably longer duration.
Gladstone et al. [47]	572	RCT	30 days of event-triggered loop recorder vs. 24 h ECG	AF was detected in 16.1% of patients with the event-triggered loop recorder compared to 3.2% of patients with 24 h ECG monitoring (*p* < 0.001). Episodes of AF spanning 150 s or longer were recorded in 9.9% of patients with event-triggered recorders compared to 2.5% of those with standard 24 h ECG monitoring (*p* < 0.001). These differences had clinical implications as 18.6% of patients in the loop recorder group had commenced anticoagulant therapy compared to 11.1% of those in the 24 h ECG monitoring group (*p* = 0.01).
Sanna et al. [48]	441	RCT	6 months of ILR vs. conventional follow-up (ECG assessment at follow-up visits, with exact protocol at the discretion of each site)	AF was detected in 8.9% of patients with an ILR by 6 months compared to 1.4% in patients receiving conventional follow-up following a cryptogenic stroke (HR 6.4 [95% CI 1.9 to 21.7], *p* < 0.001). By 12 months, AF was detected in 12.4% of patients with an ILR compared to 2.0% in those with conventional follow-up (HR 7.3 [95% CI 2.6 to 20.8], *p* < 0.001). In the patients followed up for 36 months, these rates grew to 30% and 3.0% respectively (HR 8.8 [95% CI 3.5 to 22.2], *p* < 0.001).

Abbreviations: AF = atrial fibrillation, HR = hazard ratio, RR = relative risk, CI = confidence interval, ECG = electrocardiogram, ILR = implantable loop recorder, ELR = external loop recorder, MCOT = mobile cardiac outpatient telemetry, RCT = randomised controlled trial, USD = United States dollar, TIA = transient ischemic attack.

**Table 2:** Meta-analyses comparing outcomes in atrial fibrillation patients prescribed different types of antithrombotic medications.

Study	Number of Patients (n)	Study Design	Treatment(s)	Outcomes (Primarily Regarding the Incidence of Stroke)	Haemorrhagic Adverse Events
Carnicelli et al. [59]	71,683	Meta-analysis	Standard-dose DOACs vs. lower-dose DOACs vs. warfarin	Relative to warfarin, standard-dose DOACs were linked to significant decreases in the risk of stroke or systemic embolism (HR 0.81 [95% CI 0.74–0.89]) and mortality (HR 0.92 [95% CI 0.87–0.97]). When compared to warfarin, lower-dose DOACs were not associated with a significant difference in the risk of stroke or systemic embolism (HR 1.06 [95% CI 0.95–1.19]). However, there was a significant decrease in mortality (HR 0.90, [95% CI 0.83–0.97].	Relative to warfarin, standard-dose DOACs were linked to a significant decrease in the risk of intracranial bleeding (HR 0.45 [95% CI 0.37–0.56]) but not in the risk of major bleeding (HR 0.86, 95% CI [0.74–1.01]). On the other hand, when compared to warfarin, lower dose DOACs were associated with a lower risk of both intracranial bleeding (HR 0.28 [95% CI 0.21–0.37]) and major bleeding (HR 0.63 [95% CI 0.45–0.88]).
Erdem et al. [60]	73,122	Meta-analysis	DOACs vs. warfarin	Compared to warfarin, there was a decreased risk of stroke or systemic embolism when taking DOACs in patients ≥ 75 years old (RR 0.57 [95% CI 0.42–0.76]) and patients < 75 years old (RR 0.74, 95% CI [0.43, 1.27]). This was statistically significant for ages ≥75 years but not ages <75 years.	Compared to warfarin, there was a significantly lower risk of major bleeding in patients taking DOACs who were ≥75 years old (RR 0.74 [95% CI 0.63–0.87]) as well as in those <75 years old (RR 0.64 [95% CI 0.44–0.93]).
Zeng et al. [61]	835,520	Meta-analysis	DOACs vs. warfarin	Relative to warfarin, DOACs were associated with a significantly lower risk of ischemic stroke (HR 0.79 [95% CI 0.71–0.87]) and mortality (HR 0.90 [95% CI 0.84–0.96]).	Relative to warfarin, DOACs were associated with a significantly lower risk of intracranial bleeding (HR 0.58 [95% CI 0.52–0.65]) and major bleeding (HR 0.79 [95% CI 0.64–0.97]) but no significant decrease in the risk of gastrointestinal bleeding (HR 0.97 [95% CI 0.73–1.29]).
Tereshchenko et al. [62]	96,017	Meta-analysis	Aspirin vs. VKAs vs. DOACs vs. placebo	After adjusting for other variables, treatment with VKAs and DOACs led to significantly lower rates of stroke or systemic embolism compared to placebo. However, the odds were not significantly lower for patients taking aspirin compared to placebo (aOR 0.77 [95% CI 0.53–1.11]).	After adjusting for other variables, there was no significant difference in the rates of major bleeding between treatments with aspirin, VKAs, and DOACs.

Abbreviations: AF = atrial fibrillation, DOAC = direct-acting oral anticoagulant, RR = relative risk, CI = confidence interval, HR = hazard ratio, OR = odds ratio, aOR = adjusted odds ratio, VKA = vitamin K-antagonists.

**Table 3:** Outcomes in meta-analyses following intravenous thrombolysis in ischemic stroke patients with atrial fibrillation.

Study	Number of Patients (n)	Number of Patients with AF and AF Prevalence (%)	Study Design	Functional Outcomes	Mortality	sICH
Hu & Ji [110]	8509	2125 (24.97%)	Meta- analysis	AF patients reported significantly lower odds of favourable functional outcomes (90-day mRS ≤ 2) following IVT compared to non-AF patients (OR 0.55 [95% CI 0.43–0.70], *p* < 0.001). Comparing AF patients who received IVT with AF patients who did not, there was no significant difference in the odds of favourable functional outcomes (OR 1.37 [95% CI 0.72–2.60, *p* = 0.331).	AF patients reported significantly higher odds of mortality following IVT compared to non-AF patients (OR 2.05 [95% CI 1.79–2.36], *p* < 0.001). Comparing AF patients who received IVT with AF patients who did not, there was no significant difference in mortality (OR 0.95 [95% CI 0.63–1.44], *p* = 0.813).	AF patients reported significantly higher odds of sICH compared to non-AF patients (OR 3.44 [95% CI 2.04–5.82], *p* < 0.001). The odds of sICH were significantly higher in AF patients receiving IVT compared to AF patients not receiving IVT (OR 3.44 [95% CI 2.04–5.82], *p* < 0.001).
Yue et al. [118]	14,801	3432 (23.19%)	Meta- analysis	AF patients were significantly less likely to experience favourable outcomes (90-day mRS ≤ 2) following IVT (OR 1.95 [95% CI 1.33–2.85], *p* = 0.001).	AF patients had significantly higher odds of mortality 90 days following IVT (OR 2.13 [95% CI 1.68–2.70], *p* < 0.001).	The odds of sICH were significantly higher amongst AF patients (OR 1.28 [95% CI 1.08–1.52], *p* = 0.006).
Patel & Bhaskar [122]	39,650	11,163 (28.15%) ^a^	Meta-analysis	AF patients had significantly lowers odds of favourable functional outcomes (90-day mRS ≤ 2) at 90 days following IVT (OR 0.512 [95% CI 0.376–0.696], *p* < 0.001)	AF was associated with significantly higher odds of sICH following IVT (OR 1.690 [95% CI 1.400–2.039], *p* = 0.851).	AF was associated with significantly higher odds of 90-day mortality following IVT (OR 1.799 [95% CI 1.218–2.657], *p* = 0.003)

Abbreviations: AF = atrial fibrillation, IVT = intravenous thrombolysis, sICH = symptomatic intracerebral haemorrhage, mRS = modified Rankin Scale, OR = odds ratio, CI = confidence interval, NIHSS = National Institute of Health Stroke Scale. ^a^ This value refers to the crude prevalence of AF. Notably, the meta-analysis by Patel & Bhaskar [122] included some studies that had data on the prevalence of AF post-IVT but not on the impact of AF on clinical outcomes, so the number of patients with data on outcomes following IVT was lower than the total number of patients.

**Table 4:** Outcomes in meta-analyses following endovascular thrombectomy in ischemic stroke patients with atrial fibrillation.

Study	Number of Patients (n)	Number of Patients with AF and AF Prevalence (%)	Study Design	Functional Outcomes	Mortality	sICH
Kobeissi et al. [107]	6131	2305 (37.60%)	Meta-analysis	No significant difference in the odds of functional independence (90-day mRS ≤ 2) between patients with AF and those without AF (OR 0.72 [95% CI 0.47–1.10], *p* = 0.13). Confounders were not adjusted for. However, following sensitivity analysis, the rate of functional independence was significantly lower for AF patients (OR 0.65 [95% CI 0.52–0.81], *p* < 0.001).	Mortality was significantly higher in patients with AF (OR 1.47 [95% CI 1.12–1.92], *p* = 0.005).	No significant difference in the odds of sICH between AF patients compared to non-AF patients (OR 1.05 [95% CI 0.84–1.31], *p* = 0.68).
Patel & Bhaskar [122]	21,148	8857 (41.88%) ^a^	Meta-analysis	There was no significant association between AF and favourable functional outcomes (90-day mRS ≤ 2) following EVT (OR 0.826 [95% CI 0.651–1.049], *p* = 0.117).	There was no significant association between AF and sICH following EVT (OR 0.982 [95% CI 0.815–1.184], *p* = 0.851).	There was no significant association between AF and 90-day mortality at post-EVT (OR 1.236 [95% CI 0.969–1.578], *p* = 0.088).
Smaal et al. [104]	1351	447 (33.09%)	Meta-analysis	After adjusting for other factors, there was no significant association between favourable functional outcomes (90-day mRS ≤ 2) and AF status (aOR 1.14 [95% CI 0.87–1.51], *p* = 0.337).	There was no significant association between AF and 90-day mortality (aOR 1.14 [95% CI 0.83–1.57], *p* = 0.410).	There was no significant association between AF and sICH (aOR 0.57 [95% CI 0.3–1.07], *p* = 0.082).

Abbreviations: AF = atrial fibrillation, sICH = symptomatic intracerebral haemorrhage, EVT = endovascular thrombectomy, IVT = intravenous thrombolysis, OR = odds ratio, aOR = adjusted odds ratio, CI = confidence interval, mRS = Modified Rankin Scale. ^a^ This value refers to the crude prevalence of AF. Notably, the meta-analysis by Patel and Bhaskar [122] included some studies that had data on the prevalence of AF post-EVT but not on the impact of AF on clinical outcomes, so the number of patients with data on outcomes following EVT was lower than the total number of patients.

**Table 5:** Outcomes following bridging therapy in ischemic stroke patients with atrial fibrillation.

Study	Number of Patients (n)	Number of Patients with AF and AF Prevalence (%)	Study Design	Functional Outcomes	Mortality	sICH
Loo et al. [131]	705	314 (44.54%)	Retrospective	For patients with AF, there was no significant difference in the likelihood of favourable functional outcomes (90-day mRS ≤ 2) between those receiving bridging therapy compared to those treated with EVT alone (35.0% vs. 33.3%, *p* = 0.761). However, for patients without AF, the rate of favourable functional outcomes was significantly higher in those receiving bridging therapy compared to those treated with EVT alone (45.2% vs. 23.7%, *p* < 0.001).	For patients with AF, there was no significant difference in the likelihood of mortality between those receiving bridging therapy compared to those treated with EVT alone (11.9% vs. 14.5%, *p* = 0.631). For patients without AF, there was no significant difference in the likelihood of mortality between those receiving bridging therapy compared to those treated with EVT alone (11.7% vs. 14.5, *p* = 0.559).	For patients with AF, there was no significant difference in the likelihood of sICH between those receiving bridging therapy compared to those treated with EVT alone (11.0% vs. 7.7%, *p* = 0.323). For patients without AF, there was no significant difference in the likelihood of sICH between those receiving bridging therapy compared to those treated with EVT alone (12.8% vs. 13.9%, *p* = 0.765).
Akbik et al. [132]	6461	2311 (35.77%)	Retrospective	In non-AF patients, treatment with bridging therapy was associated with a significantly increased likelihood of favourable functional outcomes (90-day mRS ≤ 2) compared to receiving EVT alone (aOR 1.29 [95% CI 1.03–1.60], *p* = 0.025). However, in AF patients, there was no significant association between bridging therapy and favourable functional outcomes (aOR 1.28 [95% CI 0.94–1.74], *p* = 0.11).	Comparing AF patients treated with bridging therapy to AF patients treated with EVT alone, there was no significant difference in mortality (27.3% vs. 25.7%, *p* = 0.593).	There were significantly elevated odds of sICH or parenchymal haematoma type 2 in AF patients who were treated with bridging therapy compared to those treated with EVT alone (aOR 1.66 [95% CI 1.07–2.57], *p* = 0.024).
Mujanovic et al. [133]	2941	1347 (45.80%)	Retrospective	Treatment with bridging therapy was associated with a significantly higher likelihood of favourable functional outcomes (90-day mRS ≤ 2) compared to EVT alone (aOR 1.61 [95% CI 1.24–2.11], *p* < 0.001). There was no significant association between AF and favourable functional outcomes following bridging therapy (aOR 0.98 [95% CI 0.66–1.46], *p* = 0.924).	There was no significant difference in the likelihood of mortality between AF patients treated with bridging therapy compared to EVT alone (21.6% vs. 28.1%, *p* = 0.038).	There was no significant association between AF and sICH following bridging therapy (aOR 1.37 [95% CI 0.67–2.83], *p* = 0.390).

Abbreviations: AF = atrial fibrillation, EVT = endovascular thrombectomy, sICH = symptomatic intracerebral haemorrhage, aOR = adjusted odds ratio, CI = confidence interval, mRS = Modified Rankin Scale. Impact of atrial fibrillation on outcomes after bridging therapy.

## Data Availability

The original contributions presented in the study are included in the article; further inquiries can be directed to the corresponding author.

## Abbreviations

IVT	Intravenous thrombolysis
EVT	Endovascular thrombectomy
AIS	Acute ischemic stroke
AF	Atrial fibrillation
TIA	Transient ischemic attack
ECG	Electrocardiogram
OAC	Oral anticoagulant
ICM	Insertable cardiac monitor
ECM	External cardiac monitor
PPG	Photoplethysmography
VKA	Vitamin K antagonist
DOAC	Direct-acting oral anticoagulant
HT	Haemorrhagic transformation
AHA	American Heart Association
ASA	American Stroke Association
RCT	Randomised controlled trial
KAF	Known atrial fibrillation
AFDAS	Atrial fibrillation diagnosed after stroke
OR	Odds ratio
mRS	Modified Rankin Scale
sICH	Symptomatic intracerebral haemorrhage
INR	International normalised ratio
NIHSS	National Institutes of Health Stroke Scale
BT	Bridging therapy
CHF	Congestive heart failure
LAE	Left atrial enlargement
MCOT	Mobile cardiac outpatient telemetry
